# Robust near real-time estimation of physiological parameters from megapixel multispectral images with inverse Monte Carlo and random forest regression

**DOI:** 10.1007/s11548-016-1376-5

**Published:** 2016-05-03

**Authors:** Sebastian J. Wirkert, Hannes Kenngott, Benjamin Mayer, Patrick Mietkowski, Martin Wagner, Peter Sauer, Neil T. Clancy, Daniel S. Elson, Lena Maier-Hein

**Affiliations:** Computer-Assisted Interventions, German Cancer Research Center, Heidelberg, Germany; Department for General, Visceral and Transplantation Surgery, Heidelberg University Hospital, Heidelberg, Germany; Department of Gastroenterology, Toxicology and Infectious Diseases, University Hospital Heidelberg, Heidelberg, Germany; Hamlyn Centre for Robotic Surgery, Institute of Global Health Innovation, Imperial College London, London, UK; Department of Surgery and Cancer, Imperial College London, London, UK

**Keywords:** Multispectral imaging, Oxygenation, Inverse Monte Carlo, Regression, Random forest, Anastomosis, Perfusion

## Abstract

**Purpose:**

Multispectral imaging can provide reflectance measurements at multiple spectral bands for each image pixel. These measurements can be used for estimation of important physiological parameters, such as oxygenation, which can provide indicators for the success of surgical treatment or the presence of abnormal tissue. The goal of this work was to develop a method to estimate physiological parameters in an accurate and rapid manner suited for modern high-resolution laparoscopic images.

**Methods:**

While previous methods for oxygenation estimation are based on either simple linear methods or complex model-based approaches exclusively suited for off-line processing, we propose a new approach that combines the high accuracy of model-based approaches with the speed and robustness of modern machine learning methods. Our concept is based on training random forest regressors using reflectance spectra generated with Monte Carlo simulations.

**Results:**

According to extensive *in silico* and in vivo experiments, the method features higher accuracy and robustness than state-of-the-art online methods and is orders of magnitude faster than other nonlinear regression based methods.

**Conclusion:**

Our current implementation allows for near real-time oxygenation estimation from megapixel multispectral images and is thus well suited for online tissue analysis.

## Introduction

Monitoring oxygenation and blood volume fraction ($v_{\text {hb}}$) is highly relevant for assessing the success of surgical treatments. One example are organ transplants. It is important to determine whether or not the transplanted organ is properly reperfused with oxygenated blood. The same applies to the example of colorectal surgery, where the integrity of a bowel anastomosis is largely dependent on adequate bowel perfusion [17]. For example, a European multicentered analysis of oncological and survival outcomes following anastomotic leakage after rectal cancer surgery showed an increased rate of 90-day postoperative mortality and morbidity from 1.3–1.9 % in patients with no anastomotic leakage to 5.8–8.9 % in patients with anastomotic leakage. In addition, the 5-year disease-free survival rate decreased from 66.9 % in patients without anastomotic leakage to 60.6 % in patients with anastomotic leakage [5].

As tissue perfusion and oxygenation cannot be accurately measured by the human eye, the automatic analysis of multispectral image data for the quantification of these and other important tissue parameters in laparoscopy has recently gained attention [3, 12, 13]. Multispectral images can be regarded as a generalization of classical RGB images. Instead of only three colors, they store an arbitrary number of images, each one corresponding to one recorded spectral band. Unlike RGB, these spectral bands are usually narrow and do not overlap, thus encoding more specific information. Each multispectral pixel can be thought of as reflectance measurement at different spectral bands. This reflectance measurement changes with the constitution of the underlying tissue, therefore containing information about physiological parameters such as oxygenation *s* or the blood volume fraction per unit volume $v_{\text {hb}}$ [8].

Deciphering this information, i.e., estimating the molecular tissue composition on the basis of multispectral images, remains challenging. Most systems for live in vivo multispectral imaging [3, 10, 12] use linear estimation approaches based on the modified Beer–Lambert law [15]. While being fast, the method is often based on the assumptions that light travels an equal pathlength in the tissue regardless of wavelength and that scattering is constant. These and similar assumptions do not hold up in real tissue. Red light penetrates the tissue deeper than blue light, because blood absorbs less in higher wavelengths. The scattering is also changing, e.g., dropping by about 20 % from 500 to 620 nm within the bowel [3, 9]. In the related fields of skin analysis as well as *ex vivo* tissue analysis, numerous methods for off-line processing of multispectral images using model-based approaches have been proposed. These rely on a tissue model to simulate labeled pairs of reflectance images and the corresponding physiological parameters. Subsequently, regression approaches such as Newton–Raphson [14], genetic algorithms [6] or support vector regression [19] are used for inverting the simulated spectra. The drawback of these methods is that they are too slow for near real-time estimation of tissue parameters when dealing with high-resolution multispectral images and are thus not suited for application during surgery or interventional procedures.

**Fig. 1:**
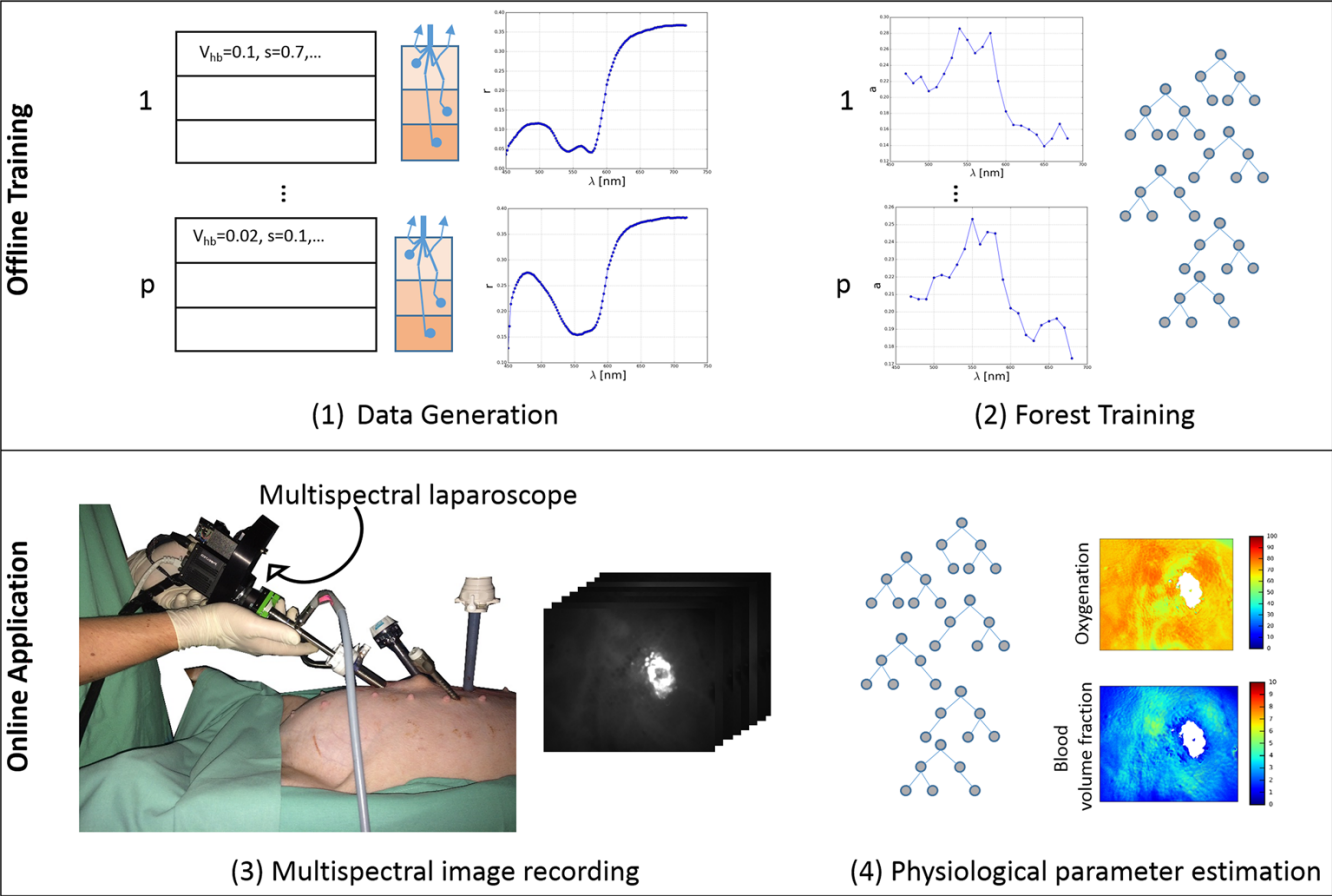
Overview of our approach. **a** Samples are drawn from our *n*-layered tissue model. Monte Carlo simulations are performed to evaluate the expected reflectance spectrum for each tissue sample. **b** The created spectra are adapted to fit the detection wavelengths of the multispectral imaging system. Noise is added, and the data are normalized and transformed to absorption. The normalized data and the physiological parameters coming from the tissue model are used to train a random forest regressor. **c** Our custom-built multispectral laparoscope is used to acquire multispectral images during interventions. Each pixel in these images corresponds to one reflectance measurement. **d** The regressor trained in (**b**) is used to estimate, e.g., oxygenation and blood volume fraction for each pixel in the multispectral image

To overcome these issues, we present the first approach to rapid estimation of physiological parameters that combines the accuracy of model-based approaches with the speed of state-of-the-art machine learning algorithms. Our method involves training a random forest regressor with large amounts of data generated with Monte Carlo (MC) simulations.

## Methods

Our approach comprises two main steps: generating the training data with a physical model and MC simulations (Sect. “Forward model for training data generation”) and using the simulated training data to train a machine learning-based regressor in Sect. “Inversion by random forest regression”. The complete process is visualized in Fig. 1.

### Forward model for training data generation

Inspired by the typical layer structure of the colon and other epithelial tissues, we model tissue inspected during minimally invasive surgery as *n*-layered structures. We characterize each layer *l* by a set of tissue properties: $\mathbf {l} = \{ v_{\text {hb}}, s, a_{\text {mie}}, b, g, n, d\}$, where the parameters describe the following:

- $v_{\text {hb}}$: blood volume fraction
- *s*: the ratio of oxygen-bound hemoglobin to total hemoglobin, also referred to as oxygenation
- $a_{\text {mie}}$: a parameter quantifying the amount of scattering
- *b*: the scattering power, a term which characterizes exponential wavelength dependence of the scattering
- *g*: anisotropy factor, characterizes the directionality of scattering
- *n*: the refractive index
- *d*: the layer thickness

the optical and physiological parameters $a_{\text {mie}}$, *b*, *g*, $v_{\text {hb}}$ and *s* influence the optical absorption and scattering coefficients. As in [8], the absorption coefficient $\mu _a$ at wavelength $\lambda$ is calculated by

1 $$\begin{aligned} \mu _a(v_{\text {hb}}, s, \lambda )= & {} v_{\text {hb}} c_{\text {hb}}(s \epsilon _{\text {HbO2}}(\lambda ) \nonumber \\&+ (1-s)\epsilon _{\text {Hb}}(\lambda )) \hbox {ln} (10) \end{aligned}$$

where $\epsilon _{\text {HbO2}}$ and $\epsilon _{\text {Hb}}$ are the molar extinction coefficients of oxygenated and de-oxygenated hemoglobin^1^ and $c_{\text {hb}}$ is the molar concentration of hemoglobin in human blood. We assume hemoglobin, which is the oxygen transporter in human blood, to be the only notable absorber [9]. As in [4] oxygenation is assumed to be the constant for all layers. This is a reasonable assumption if the layers share a common blood supply as, e.g., in the colon [4]. The reduced scattering coefficient $\mu _s^{\prime }$ is calculated by an empirical power law (see [9])

2 $$\begin{aligned} \mu _s^{\prime }(a_{\text {mie}}, b, \lambda ) = a_{\text {mie}}\left( \frac{\lambda }{500\,\hbox {nm}}\right) ^{-b}. \end{aligned}$$

The simple form for reduced scattering was chosen, as scattering by large particles dominates in the visible spectrum. The anisotropy *g* is assumed constant over the wavelength range, and the scattering coefficient $\mu _s$ is calculated by $\mu _s(a_{\text {mie}}, b, \lambda )= \frac{\mu _s^{\prime }(a_{\text {mie}}, b, \lambda )}{1-g}$ [9].

**Table 1:** Parameter ranges for the colon tissue model

	$v_{\text {hb}}$ (%)	*s* (%)	$a_{\text {mie}}$ ($\hbox {cm}^{-1}$)	*g*	*n*	*d* ($\upmu \hbox {m}$)
$\mathbf {l}_1$	0–10 [4]	0–100	$18.9 \pm 10.2$ [9]	0.8–0.95	1.36 [4]	600–1010 [8]
$\mathbf {l}_2$	0–10 [4]	0–100	$18.9 \pm 10.2$ [9]	0.8–0.95	1.36 [4]	415–847 [8]
$\mathbf {l}_3$	0–10 [4]	0–100	$18.9 \pm 10.2$ [9]	0.8–0.95	1.38 [4]	395–603 [8]

To generate a multispectral reflectance spectrum $\mathbf {r} \in \mathbb {R}^m$ from our *n*-layer model, a function $f_{\text {sim}}$ is evaluated at *m* wavelengths $\lambda$:

3 $$\begin{aligned} r(\lambda ) = f_{\text {sim}}(\mathbf {\lambda }, \mathbf {l}_1, \ldots , \mathbf {l}_n). \end{aligned}$$

For this publication, a multilayered MC (MCML) approach was chosen for evaluation of $f_{\text {sim}}$, because MC models are widely considered to be the gold standard for calculating how light travels through biological tissue. Aside from being more accurate than, e.g., the diffusion approximation or the modified Beer–Lambert law, it is easy to configure and flexible. The MC tissue optics simulation irradiates multilayered tissue with photon packets [20]. Depending on the layers’ properties, the photons will be probabilistically reflected, scattered and absorbed. Among other attributes, the photons reflected at the tissue surface due to (possibly multiple) scattering events can then be measured.

### Inversion by random forest regression

In the last section, we described how to create one single reflectance spectrum given one instance of our tissue model. To train machine learning-based methods, however, a large amount of samples has to be available. Thus, a range of layer parameters, which are plausible for the tissue we want to observe, has to be defined and *p* reflectance spectra for instances of our tissue model within these ranges have to be generated. Possible ranges for the different parameters are provided in Table 1. This yields the raw data for the regressor.

Before training the regressor, the spectra need to be adapted to the imaging system and normalizations to account for variabilities in real-world scenarios have to be applied. For each filter *k* in the multispectral imaging system, the filter’s transmission spectrum $b_k(\lambda )$ is taken into account to calculate the reflectance measured by the imaging system $r_k$

4 $$\begin{aligned} r_k = w + \sum _{\lambda _{\text {min}}}^{\lambda _{\text {max}}}b_k(\lambda )r(\lambda ), \end{aligned}$$

where *w* represented zero-mean Gaussian noise, which models nuisance factors as, e.g., camera noise.

Our method is aimed for in vivo application. Thus, it is necessary to account for constant multiplicative changes in reflection. These changes can, e.g., be caused by differences in distance or angle of the camera to the tissue and the internal scaling of reflection to values measured by the camera [4]. By applying the *l*1 norm to the reflection values, these changes are easily canceled out: $r_k^{\prime } = \frac{c r_k}{c\sum _{j} r_j}$. Transformation to absorption by applying $-$log and further normalization by the *l*2 norm results in $\mathbf {a}_{\{1\ldots p\}}$.

The vectors $\mathbf {a}_{\{1\ldots p\}}$ along with their corresponding oxygenations and blood volume fraction in the first layer were used as training inputs for a random forest (RF) regressor. Random forest regressors [2] average an ensemble of random regression trees. The RF regressor was chosen due to its rapid evaluation speed and general good performance.

## Experiments and results

In a set of *in silico* (Sect. “*In silico* quantitative validation”) and in vivo (Sect. “In vivo qualitative analysis”) experiments, we investigated the accuracy, robustness and run-time of our new method compared to the widely applied linear Beer–Lambert regression model described, e.g., in [3] as the baseline method.

### Data generation

Anastomosis success verification is a potential application for multispectral imaging. We defined a tissue model for colon tissue with the parameter values summarized in Table 1. The values were chosen to mimic colonic tissue viewed from the pneumoperitoneum with values chosen from the literature when available. Values were drawn randomly from these parameter ranges. The scattering power *b* was set to 1.286, a mean value for soft tissues [9]. The molar hemoglobin concentration $c_{\text {hb}}$ was set to $120\,\hbox {gL}^{-1}$, a typical value in the colon as opposed to $150\,\hbox {gL}^{-1}$ in general human tissue [8]. This parameter scales the blood volume fraction estimation result, as $c_{\text {hb}}$ and $v_{\text {hb}}$ cannot be distinguished by optical means (see Eq. 1).

In our experiments, we evaluated the MC simulation in the [450, $720\,\hbox {nm}$] interval in $2\,\hbox {nm}$ steps. The open-source GPU-MCML [1] implementation was used as simulation framework. The number of photon packets fired was set to $10^6$ in all simulations, and the diffuse reflectance was taken as the reflectance value.

### Random forest parameters

The RF parameters were determined by fivefold cross-validation and grid search on the training data, varying the maximum forest depth from three to ten in increments of one. The minimum samples per leaf were evaluated for 1, 5, 10, 20 and 100. The number of forests was set to ten to keep the computational effort manageable. Experiments with larger and deeper forests showed no change in performance. The best forest parameter setting thus determined featured a depth of nine and a minimum of ten samples per leaf. These parameters were used in the subsequent experiments.

### *In silico* quantitative validation

In the *in silico* experiments, we investigated how factors like the number of samples, noise and domain switch influence the regression result.

If not mentioned otherwise, all experiments were conducted with 15000 training samples generated from the model specified in Sect. “Data generation” and tested with a separate set of 5000 samples. The noise was varied by modifying the signal-to-noise ratio SNR: $w=\frac{r_k}{\text {SNR}}$. If not mentioned otherwise, the SNR was set to ten. To simulate a typical multispectral camera, the spectrum was parsed in $10\,\hbox {nm}$ increments from 470–680 nm. For each of these central wavelengths, a $10\,\hbox {nm}$ sliding average simulated the filter bandwidth $b_k(\lambda )$.

**Fig. 2:**
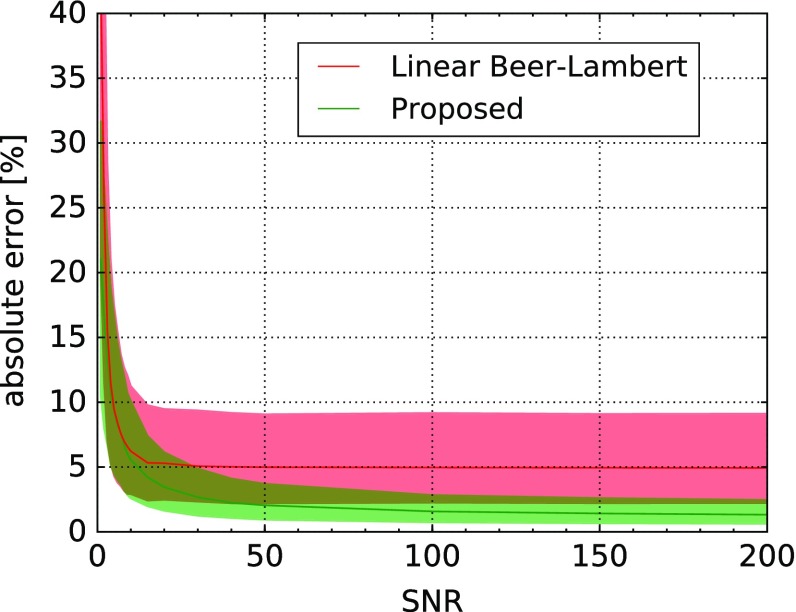
Error if training and testing data are based on the same noise distribution: zero-mean Gaussian noise with standard deviation corresponding to the SNR. Depicted is the median absolute error in oxygenation estimation and corresponding interquartile ranges as a function of the amount of noise for baseline and the proposed random forest-based method with 15,000 training samples

**Fig. 3:**
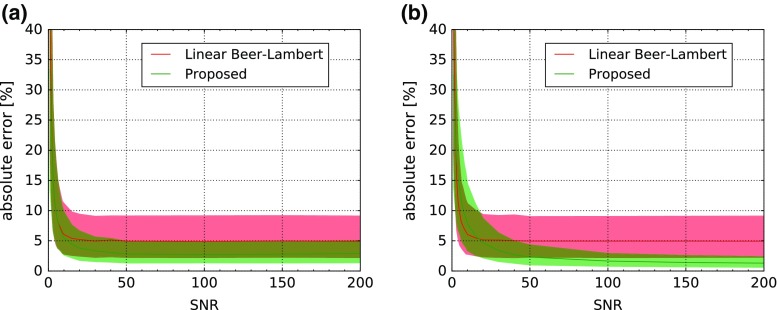
Effect of unequal test and training noise. **a** Low SNR for training, varying testing noise. **b** High SNR for training, varying testing noise. Depicted is the median absolute error in oxygenation estimation and corresponding interquartile ranges as a function of the amount of noise for baseline and the proposed random forest-based method with 15,000 training samples

*Dependency on noise* In these experiments, we varied the Gaussian noise of Eq. 4. First we investigated the performance of the classifier while adding noise of the same distribution for training and testing data (Fig. 2). For SNRs above ten, our approach outperforms the linear Beer–Lambert approach. Since the determination of noise is not trivial, we also investigated the effect of differing training and testing noise (Fig. 3). Even under these conditions, our approach showed lower errors for SNRs above twenty.

As $v_{\text {hb}}$ cannot be measured by the baseline method, no comparison has been made here. The median absolute error stays below $1.6\pm 1.2\,\%$ regardless of noise and below $1\pm 1.1\,\%$ for SNRs larger than 10.

**Table 2:** Parameter ranges for the generic tissue model

	$v_{\text {hb}}$ (%)	*s* (%)	$a_{\text {mie}}$ ($\hbox {cm}^{-1}$)	*g*	*n*	*d* ($\upmu \hbox {m}$)
$\mathbf {l}_1$	0–100	0–100	$18.9 \pm 10.2$ [9]	0.8–0.95	1.33–1.54 [9]	0–2000
$\mathbf {l}_2$	0–100	0–100	$18.9 \pm 10.2$ [9]	0.8–0.95	1.33–1.54 [9]	0–2000
$\mathbf {l}_3$	0–100	0–100	$18.9 \pm 10.2$ [9]	0.8–0.95	1.33–1.54 [9]	0–2000

*Performance under domain switch* Knowledge about the model may not always be available. Furthermore, malignancies can change the parameter ranges. An example of such a malignancy are carcinomas which, due to angiogenesis, may show abnormally high values for $v_{\text {hb}}$. To test the effect of different parameter ranges, we generated data from a second model with the values specified in Table 2. As in the colon model oxygenation was assumed constant for all layers. As an additional constraint all layers were normalized so they totaled a maximum of 2 mm in depth.

When training with this model, the median absolute errors of the proposed method and the baseline are the same. The proposed method’s 75 % quartile error is 3 % higher and the 25 % quartile error is 0.7 % lower than the baseline.

*Accuracy and run-time compared to state-of-the-art regression methods* We compared Python implementations (scikit-learn) of the proposed RF regressor with support vector regression (SVR) and k-nearest neighbors (k-NN) regression. Using grid search as in Sect. “Random forest parameters” we determined the best parameters for the SVR to be the radial basis function (RBF) kernel with $C=100$ and $\gamma =10$. Five neighbors were used for k-NN as in [18], and the algorithm parameter was set to auto. The experiment was conducted on a Intel Core™i7 CPU@3.20GHzx12 machine

**Fig. 4:**
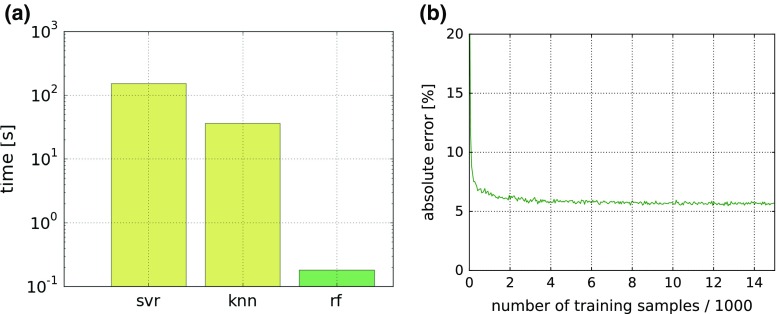
Timing results and evaluation of necessary training samples. **a** Mean time for estimating oxygenation for a multispectral imaging stack of dimension $1000 \times 1000 \times 8$, where eight is the number of spectral bands used. Compared are the proposed random forest (RF) approach, support vector regression (SVR) and k-nearest neighbors (k-NN). **b** Median absolute error in oxygenation estimation as a function of the number of training samples. The results stabilize after training with about $10^4$ samples

As can be seen in Fig. 4a, our method is at least two orders of magnitudes faster than the baseline approaches. It took 0.18 $\pm \,0.01$ s to evaluate *s* and $v_{\text {hb}}$ for a one megapixel image. The median absolute error was $5.4\,\%$ for RF, $4.8\,\%$ for SVR and $5.5\,\%$ for k-NN. The linear Beer–Lambert method evaluates one megapixel in 0.03 s.

*Number of samples* As the generation of training data is time-consuming even with the graphics processing unit (GPU) accelerated MC simulation used in this publication, we analyzed how much data would be really necessary for training the regressor. Figure 4b shows stabilization after training with about $10^4$ samples.

**Fig. 5:**
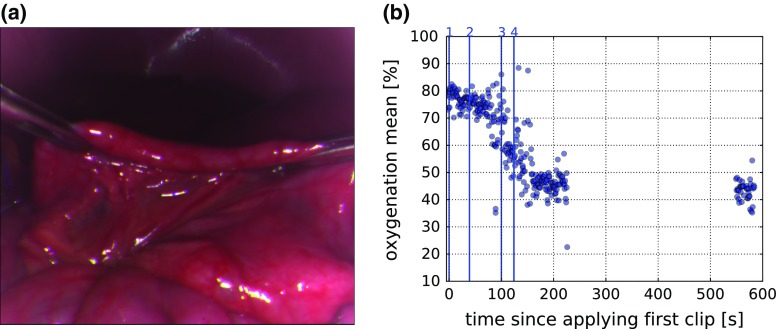
Qualitative validation of the proposed method in a porcine small bowel (**a**). The plot (**b**) shows the mean oxygenation in the small bowel segment as a function of time. The *vertical lines* show the time points at which the three vessels were clipped. The first two clips were applied to the same vessel. The high variations before setting the third clip and after setting the forth clip are caused by fast camera movements. After setting the last clip, the camera was removed and reinserted about 10 min after setting the first clip

### In vivo qualitative analysis

We used a custom-built hardware setup combining a Richard Wolf (Knittlingen, Germany) laparoscope and light source with a Pixelteq (Largo, FL, USA) 5Mpix Spectrocam. The filters were determined with the method from [21] with central wavelengths of 470, 480, 511, 560, 580, 600, 660 and 700 nm. The full width at half maximum of the bands is 20 nm, except for the 480 nm band where it is 25 nm. The acquisition of one multispectral image stack takes 400 ms. We downsampled the images to one-fourth of the original size. This leads to a resolution of $1228 \times 1029$ pixels, which is similar to modern laparoscopic HD optics. No further post-processing in the form of image registration or Gaussian smoothing as in [3] was performed. The multispectral images were divided by a recorded flatfield [11] to make them independent of the light source illumination, laparoscope optics and quantum efficiency of the camera. Before division both multispectral image and flatfield were subtracted by the camera’s dark current [11].

**Fig. 6:**
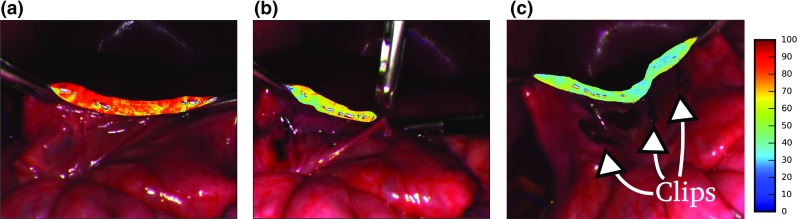
Visualization of small bowel oxygenation estimation. The clipped small bowel segment was segmented to estimate mean oxygenation. The *color bar* shows oxygenation in percent. **a** Before clipping. **b** Setting of the fourth clip. **c** Nine minutes after applying first clip

**Fig. 7:**
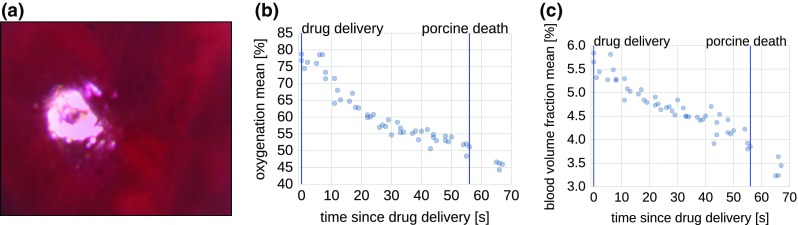
Qualitative validation of the proposed method in a porcine liver (**a**). The plots show the mean oxygenation (**b**) and blood volume fraction (**c**) after lethal drug delivery as a function of time. Specular pixels were excluded by simple thresholding

A rough estimate of the camera’s SNR was determined by calculating the mean intensities for each band, using all images acquired during one experiment. We subtracted the mean intensities by the dark current and divided this result by a camera noise estimate for the determined mean intensities. This leads to a SNR range of 29 for the 470 nm band to 47 for the 660 nm band. The differences in SNR are mainly caused by the hemoglobin absorption, light source spectrum and camera quantum efficiency. To train the random forest, the SNR was set to ten to account for variations caused by camera-tissue movements. This is also motivated by the *in silico* results, which indicated that the method is robust to conservatively estimated errors.

In a first experiment, we stopped the blood supply of a small bowel segment in a porcine model by clipping three vessels connected to the segment (see Fig. 6c). To simulate real operating conditions, we recorded a multispectral video of the breathing swine while moving the camera and the instruments in a clinically realistic manner. Mean oxygenation of the clipped bowel segment was determined by manually segmenting the bowel segment and excluding specular regions by thresholding. A sharp drop in oxygenation was detected after clipping. Figure 5b visualizes the estimation result derived from all 315 multispectral image stacks of the recorded video (Fig. 6).

In a second experiment, we recorded images of a porcine liver directly after lethal drug delivery. Mean oxygenation was determined over all image pixels except for specular regions, which were excluded by thresholding. A steady drop of oxygenation and blood volume fraction was measured as shown in Fig. 7, which shows the estimation results for all 51 recorded image stacks.

## Discussion

In this contribution, we developed a machine learning-based method for deriving physiological parameters from multispectral images. It is based on generating labeled training data using a physical model and highly accurate MC simulations. Random forests are used to invert the MC spectra and derive oxygenation and blood volume fraction for every pixel in the recorded image.

The estimation of these parameters is of relevance for monitoring the success of operations like colectomies or organ transplants. The method could also be applied in minimally invasive cancer screenings such as colonoscopies. By detecting changes in blood volume fraction and oxygenation, it could help in detecting polyps and flat adenomas. To guarantee practical applicability in these scenarios, special emphasis was given to developing a fast, but powerful, method.

Unlike the compared state-of-the-art method, the presented approach is capable of estimating both oxygenation and blood volume fraction. Due to the underlying MC framework, it is not restricted to assumptions as constant light penetration depth and scattering losses. In the following, we discuss our forward model and the proposed inversion technique as well as our results.

*Forward model*

Oxygenated and de-oxygenated hemoglobin were chosen as sole absorbers in our model because they are the only notable absorbers of visible light in human tissue besides melanin, which, however, is mainly contained in the skin [9] and thus irrelevant for minimally invasive surgical applications. Note that our model allows for straightforward integration of further absorbers (if necessary) by modification of Eq. 1. Modification of the tissue composition requires investigation as to whether the generated spectra can still be inverted, e.g., by using the regression techniques presented in this paper.

The value for anisotropy is hard to measure and is not well understood for human tissues, as experiments and the results from theoretical analysis by Mie theory do not coincide [9]. Most experiments come to the conclusion that human tissue is strongly forward scattering with quite high levels of *g* in the visible range [9]. Therefore, we modeled the anisotropy to be in a range that covers most of the experimental values depicted in [9].

*Inversion by random forest regression* Random forest regressors were chosen because they are capable of near real-time regression of megapixel multispectral images. Additionally, random forest regressors are inherently multivariate and thus allow joint oxygenation and blood volume fraction estimation.

From a machine learning standpoint, the additive Gaussian noise *w* is necessary to prevent over-fitting of the regressor. The term models noise from the camera, tissue/camera movement during image acquisition and model inaccuracies. Model inaccuracies can, e.g., be the presence of additional unknown absorbers, cross talk between pixels caused by inhomogeneous tissue or tissue structures not modelable by a multilayer model. To account for the latter two, 3D MC simulations would be a viable option for future experiments. Such a setup would need careful design to both ensure the modeling of realistic tissue and be general enough to cover a relevant tissue variability.

To account for constant multiplicative illumination changes, we applied the *l*1 norm. Other normalizations proposed to account for these changes are the usage of image quotients [16] or division by the integral of the reflectance spectrum [4]. The *l*1 norm was chosen because it is more robust to noise than image quotients; the integral can be tricky to calculate in cases where the spectral bands are unevenly spaced and sparse.

The additional normalizations of transformation to absorption and further *l*2-normalization could be left out in principle because nonlinear regressors were applied. We found, however, that doing these normalizations improves the mean absolute regression errors by more than 4 % for our method. The also analyzed SVR did not necessitate the *l*2 normalization and transformation to absorption. The k-NN result dropped by 10 % when omitting normalizations.

The developed method can also be seen as an *in silico* testing stage for hardware setups. It could, for example, help in choosing the most relevant multispectral bands. Furthermore, the framework can be used to compare different inversion techniques. To this end, we made our Python framework available on GitHub.^2^

*Experimental setup* For this publication, an MCML approach was chosen to create the reflectance spectra, because MC models are widely considered to be the gold standard for calculating how light travels through biological tissue. Aside from being more accurate than, e.g., the diffusion approximation or the modified Beer–Lambert law, it is easy to configure and flexible.

Its main drawback is the time consumption. With the setup described in Sect. “Experiments and results”, the evaluation of one reflectance spectrum took about 16 s by an off-the-shelf desktop PC with a NVIDIA GeForce GTX 660 Ti graphics card. The simulation of 15,000 spectra used for training our regressor therefore took less than 3 days. Because it is only required for training, this one-time investment of three-day computation was not seen as critical.

*Experimental results* Our method outperforms the baseline method for SNRs above ten and is at least as good as the baseline for SNRs below ten. This is true even if the training noise is set to a fixed SNR of ten and the testing noise is varied. The main challenge determined by our experiments was applying the method to data from a different domain. In future works, we will therefore use data generated from a more generic model to train our regressor. To combat the introduced covariate shift, domain adaptation methods will be applied. Other advanced nonlinear methods show comparable performance but lack the rapid evaluation speed shown by our choice of random forests. The methods inverts $10^6$ spectra with more than $5\,\hbox {Hz}$. As our camera records images with $2.5\,\hbox {Hz}$, the method is fast enough for real-time processing of these images.

In both in vivo experiments, the expected drops in oxygenation and blood volume fraction have been observed. The initial value of oxygenation of 70–85 % in both experiments is in line with the literature [7]. The drop in oxygenation of the bowel is in line with the experiments performed in [3]. The drop in oxygenation of the liver after euthanasia is also expected. The blood volume fraction probably decreases due to the loss of blood pressure and subsequent drainage of blood from the liver surface due to gravity. The results on the in-vivo tissues are especially encouraging, as the regressor was trained on data tailored to colonic tissue.

We determined the camera to have SNRs ranging from 29 to 50 in our experiments. The recorded images were quite dark with mean values within the lowest percentile of the camera’s dynamic range. This can be ameliorated with a brighter light source. Additional, probably more critical noise is introduced by tissue and camera movement during image acquisition, which takes 400 ms. Alignment algorithms, noise reduction schemes tailored for multispectral images and faster camera techniques should be explored to reduce the expected noise. In future work, we will build elaborate tissue-mimicking phantoms to further validate the approach.

In conclusion, our method features both the flexibility and realism of complex model-based approaches and speed comparable to simple online methods. According to extensive *in silico* and in vivo experiments, our method is more flexible and accurate than the commonly used Beer–Lambert law-based regression and orders of magnitude faster than regression approaches developed for skin and *ex vivo* multispectral image analysis. Due to its robustness, nonlinear estimation capability and rapid execution time, there is a high potential for future application in interventional multispectral imaging.

## Footnotes

1 Values taken from http://omlc.org/spectra/hemoglobin/.

2 https://github.com/swirkert/ipcai2016.
